# The Surgical Diseases of Children, and Their Treatment by Modern Methods

**Published:** 1895-12

**Authors:** 


*The Surgical Diseases of Children, and their Treatment by Modern Methods.* By D'ARCY POWER, M.B. Pp. xvi., 548. London: H. K. LEWIS. 1895.

Practical experience and careful attention to the views of other writers—two essential qualifications for the author of any book on surgery or medicine—are well illustrated in the work before us. Its general excellence is very high, and special value attaches to certain portions of it. The mechanical portion of the work is good, though we did not expect to see so many misprints, amongst which are the following:—at page 177, line 22, for "femur" read "humerus"; at page 239, line 27, for "acromion" read "olecranon"; at page 314, line 14, for "more" read "mere"; at page 471, line 13, for "glands" read "glans."

The description of the various types of disease is good; and the book will be extremely useful to all who are interested in the study of children's diseases, especially to the busy practitioner who has not time for the investigation of original authorities. The first chapter deals with the qualifications of those who have to attend sick children, and it contains many valuable suggestions, including a description of transfusion and the method of performing the operation. The third chapter deals clearly and succinctly with the various forms of septic disease. We are pleased that the author strongly advocates the early removal of enlarged glands when a fair trial of constitutional measures has failed to give relief. We have had occasion to adopt this method of treatment a great many times; in some cases from twenty to thirty glands have been enucleated, and the wounds have generally healed by first intention, the very best results on the general health of the child being immediately apparent. The various diseases of the bones and joints are effectively dealt with, and, prefixed to the chapter on joint-disease, there are excellent diagrams, showing the relation of the synovial membranes to the epiphyses in the larger joints. The study of these relations is of great importance in considering the starting-point of disease. Arthrectomy, excision, and other operations upon the joints are fully described. Much care has been taken by the author to define the various kinds of fracture, especially those involving the joints and epiphyses. Practitioners are cautioned as to the risks of anchylosis attending such injuries, especially in the neighbourhood of the elbow. Chapter IX. contains a useful article upon "arthrodesis," the somewhat novel operation for anchylosing flail-joints, which much commends itself to our judgment. In considering the causes of genu-valgum, Mr. Power should have laid more stress upon overgrowth of the inner tuberosity of the tibia; for we have seen several cases in which the condition was due entirely to this cause.

There is an exhaustive chapter upon diseases of the ear, and the secondary troubles to which they give rise; and the surgery of the brain is well illustrated by diagrams to indicate the precise spots at which the skull should be trephined. Chapter XVII. treats of diseases of the air-passages; and includes a good article upon diphtheria, which is up to date and describes the treatment of the disease by antitoxin, by intubation, and by tracheotomy. Antitoxin, we are told, is still on its trial as a remedial measure. Hernia in its various forms is well described, and much stress is laid upon the operation for radical cure. Phymosis is so common amongst children, that the long article upon it and upon the operation of circumcision is well worth reading. We cannot, however, agree with the condemnation of dilatation of the prepuce as a treatment for phymosis, for we have frequently succeeded in curing very severe constrictions by this means. The mistake lies in dilating too rapidly, and in not drawing the prepuce back twice a day. The plan of "enucleation" is not alluded to in the treatment of subcutaneous nævi; it is very serviceable.

We think that, notwithstanding the author's apology, orthopædic surgery should receive some notice in a work upon the surgical diseases of children; and that at least a short chapter on the early treatment of talipes would be of the greatest use to general practitioners. Nearly all such cases of deformity first come under their notice, and it is not very rare to meet with cases in which the doctor has told the parents of the child that it will grow out of the clubfoot. A chapter upon the diseases of the eye, which are common in infancy and childhood, would also render the work still more complete. With these suggestions we take leave of a work the perusal of which has proved interesting and profitable. We cordially recommend it to the consideration of our professional brethren.

